# Synaptic Dysbindin-1 Reductions in Schizophrenia Occur in an Isoform-Specific Manner Indicating Their Subsynaptic Location

**DOI:** 10.1371/journal.pone.0016886

**Published:** 2011-03-01

**Authors:** Konrad Talbot, Natalia Louneva, Julia W. Cohen, Hala Kazi, Derek J. Blake, Steven E. Arnold

**Affiliations:** 1 Department of Psychiatry, Center for Neurobiology and Behavior, University of Pennsylvania, Philadelphia, Pennsylvania, United States of America; 2 MRC Centre for Neuropsychiatric Genetics and Genomics and Department of Psychological Medicine and Neurology, Cardiff University, Cardiff, Wales, United Kingdom; Nathan Kline Institute and New York University School of Medicine, United States of America

## Abstract

**Background:**

An increasing number of studies report associations between variation in *DTNBP1*, a top candidate gene in schizophrenia, and both the clinical symptoms of the disorder and its cognitive deficits. *DTNBP1* encodes dysbindin-1, reduced levels of which have been found in synaptic fields of schizophrenia cases. This study determined whether such synaptic reductions are isoform-specific.

**Methodology/Principal Findings:**

Using Western blotting of tissue fractions, we first determined the synaptic localization of the three major dysbindin-1 isoforms (A, B, and C). All three were concentrated in synaptosomes of multiple brain areas, including auditory association cortices in the posterior half of the superior temporal gyrus (pSTG) and the hippocampal formation (HF). Tests on the subsynaptic tissue fractions revealed that each isoform is predominantly, if not exclusively, associated with synaptic vesicles (dysbindin-1B) or with postsynaptic densities (dysbindin-1A and -1C). Using Western blotting on pSTG (n = 15) and HF (n = 15) synaptosomal fractions from schizophrenia cases and their matched controls, we discovered that synaptic dysbindin-1 is reduced in an isoform-specific manner in schizophrenia without changes in levels of synaptophysin or PSD-95. In pSTG, about 92% of the schizophrenia cases displayed synaptic dysbindin-1A reductions averaging 48% (p = 0.0007) without alterations in other dysbindin-1 isoforms. In the HF, by contrast, schizophrenia cases displayed normal levels of synaptic dysbindin-1A, but 67% showed synaptic reductions in dysbindin-1B averaging 33% (p = 0.0256), while 80% showed synaptic reductions in dysbindin-1C averaging 35% (p = 0.0171).

**Conclusions/Significance:**

Given the distinctive subsynaptic localization of dysbindin-1A, -1B, and -1C across brain regions, the observed pSTG reductions in dysbindin-1A are postsynaptic and may promote dendritic spine loss with consequent disruption of auditory information processing, while the noted HF reductions in dysbindin-1B and -1C are both presynaptic and postsynaptic and could promote deficits in spatial working memory.

## Introduction

Among the many genes which may promote development of schizophrenia, *DTNBP1* (dystrobrevin binding protein 1) remains among the top candidates [1], [2] and is hence among the most intensively investigated. Twenty studies on populations across the globe report significant associations between schizophrenia and one or more *DTNBP1* SNPs and/or haplotypes (cf. [3]–[6]). An increasing number of studies report that several of these *DTNBP1* risk variants are associated with severity of the positive symptoms (e.g., delusions and hallucinations) and especially the negative symptoms (e.g., flattened affect and social withdrawal) of schizophrenia [7]–[10]. Such genetic variants are also associated with severity of cognitive deficits in this disorder [3],[11]–[14]. Indeed, several *DTNBP1* risk SNPs are significantly more common in the subset of schizophrenia cases marked not only by earlier adult onset and more chronic course, but by more prominent positive and negative symptoms, as well as greater cognitive deficits [15].

There is consequently escalating interest in understanding the role of *DTNBP1* variants and of its encoded protein in pathophysiology of schizophrenia. That protein is commonly known as dysbindin and more accurately as dysbindin-1 [5]. It is the largest member of a protein family with three paralogs (dysbindin-1, -2, and -3) encoded by different genes yet sharing sequence homology in a region called the dysbindin domain [5]. Each paralog exists in multiple isoforms. Among the 16 splice variants of *DTNBP1* mRNA listed in the AceView database, there are three reference variants (NM_032122, NM_183040, and NM_183041). They appear to be the most commonly expressed transcripts given the number of cDNA clones available for their reconstruction. These three transcripts encode the isoforms we call dysbindin-1A, -1B and -1C (NP_115498, NP_898861, and NP_898862, respectively, in NCBI Entrez Protein database [5]).

How *DTNBP1* risk SNPs or haplotypes may affect dysbindin-1 is still unknown, but it is known that levels of this protein are lower in two brain areas dysfunctional in schizophrenia. A quantitative immunohistochemical study on the hippocampal formation (HF) found that more than 70% of the schizophrenia cases tested had lower presynaptic levels of dysbindin-1 compared to matched controls [16]. A more recent Western blotting study on whole-tissue lysates of dorsolateral prefrontal cortex similarly found that more than 70% of schizophrenia cases studied had lower levels of dysbindin-1 than matched controls, but limited to dysbindin-1C [17].

These findings collectively suggest that dysbindin-1 reductions in schizophrenia may be isoform specific reductions occurring in synaptic tissue. To test that hypothesis, we conducted the first Western blotting studies on synaptic dysbindin-1 in this disorder. We specifically tested synaptosomal levels of its major isoforms in the temporal lobe of schizophrenia cases compared to matched controls and the subsynaptic localization of those isoforms to interpret the results. The temporal lobe structures investigated were the HF and the posterior half of the superior temporal gyrus (pSTG). The latter consists primarily of auditory cortices: the primary (or core) auditory cortex (Brodmann, BA, area 41), a surrounding secondary auditory area (i.e., auditory belt, BA42), and adjoining auditory parabelt on the lateral wall of the pSTG (caudolateral BA 22) [18]–[21].

We focused on the HF and STG since they are among the most profoundly affected brain areas in schizophrenia, displaying diverse structural, chemical, and physiological abnormalities in neuronal assemblies [21]–[24]. Such abnormalities in the STG are well known to extend into the pSTG (cf. refs. [25]–[29]). These abnormalities are of such magnitude in the HF and STG, including primary and secondary auditory areas of the pSTG, that macroscopic volume reductions occur there in schizophrenia (cf. [21], [24], [29]–[32]) and are evident even at the time of diagnosis [31], [33], [34]. The degree of tissue shrinkage is significantly correlated with the degree of memory and executive function deficits in the case of HF volume reductions [35]–[38] and with the frequency and severity of auditory hallucinations in the case of STG volume reductions [39]–[42].

## Results

### Dysbindin-1 distribution seen immunohistochemically

**Figure 1:**
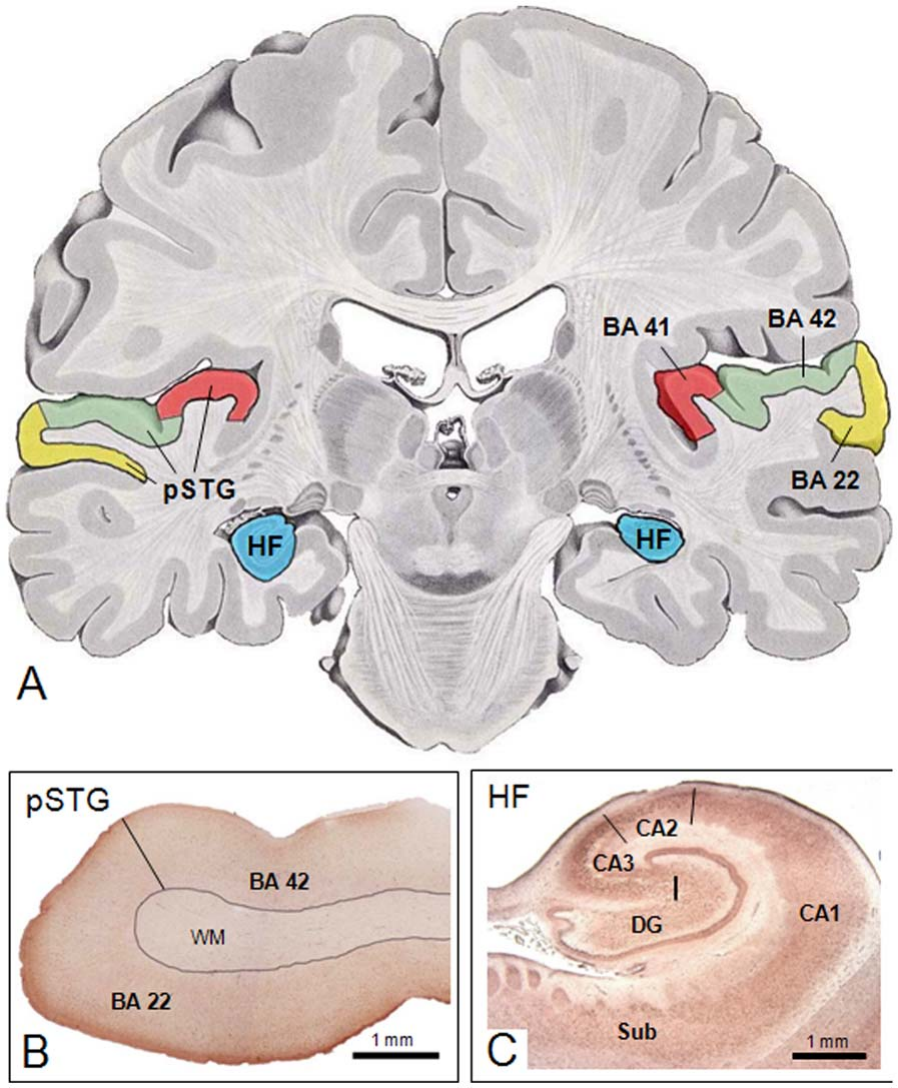
Temporal lobe areas studied: posterior superior temporal gyrus (pSTG) and intermediate rostrocaudal hippocampal formation (HF). **A**: Location of those structures mapped on a template adapted from Nieuwenhuys et al. [85]. **B** and **C**: Tissue sections from pSTG and HF, respectively, reacted immunohistochemically for dysbindin-1 with antibody PA3111. pSTG samples included BA 22 and 42. The cellular localization of dysbindin-1 in the sampled areas is shown in Figure 2.

**Figure 2:**
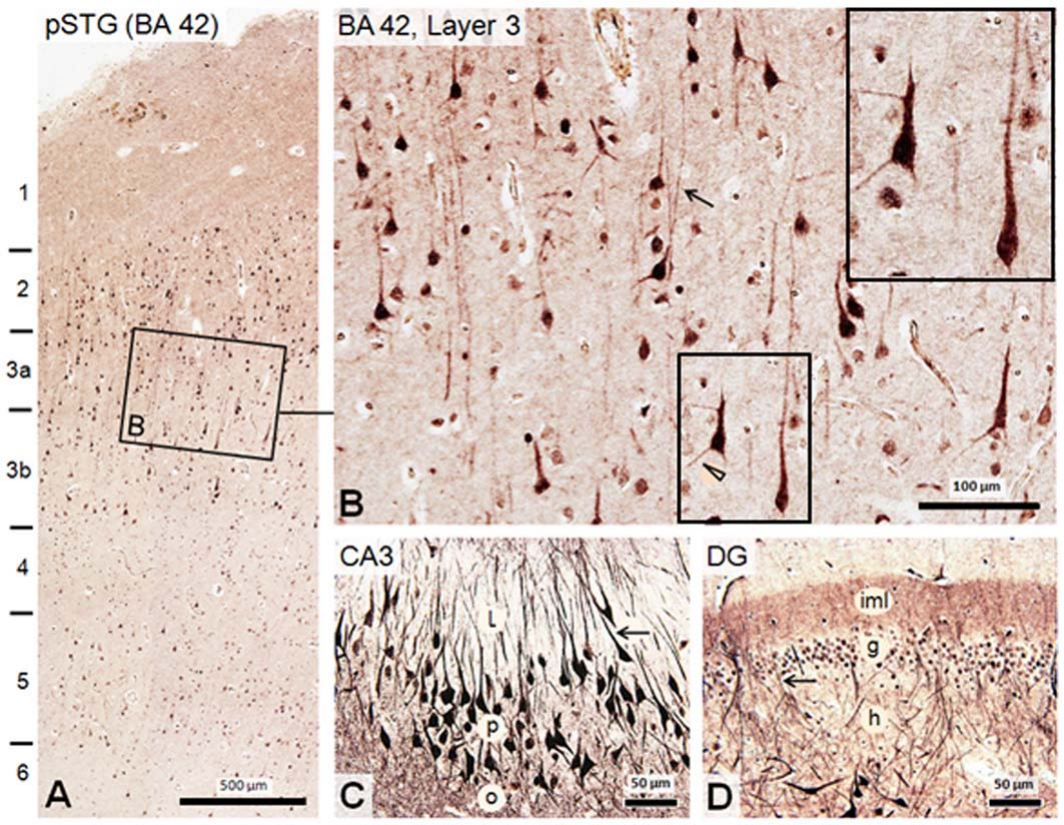
Distribution of dysbindin-1 in normal human pSTG and HF as seen immunohistochemically with PA3111. **A**: While higher neuropil concentrations of the protein are evident in the HF, dysbindin-1 is also present in neuropil of the pSTG, most notably in superficial layers 1–3. **B**: Higher magnification view of boxed area **A**, which reveals dysbindin-1 not only in neurons of Brodmann area (BA) 42, but in pyramidal cell apical and basal dendrites such as those indicated by an arrow and arrow head, respectively. Inset shows higher magnification of boxed field. The smaller, less immunoreactive cells may be interneurons. Higher dysbindin-1 levels are seen in pyramidal cells of hippocampal field CA3 (**C**) and in polymorph cells of the dentate gyrus (DG) (**D**), including dendrites (arrowed) and axon terminal fields of those cells. l, o, and p = strata lucidum, oriens, and pyramidal of CA3, respectively; g, h, iml = granule cell, hilus, and inner molecular layer of the DG, respectively.

Immunohistochemistry with our PA3111 antibody [16], [43] (Figures 1B, C and 2), which recognizes all three major isoforms of dysbindin-1, revealed that this protein is neuronal, not glial. In the pSTG, the protein was expressed in neurons and their cell nuclei in layers 2–6, but was highest in layers 2 and 3 (Figure 2A). In pyramidal cells of layer 3, the immunoreactivity spread into basal and especially apical dendrites (Figure 2B). Diffuse immunoreactivity in synaptic tissue between neuronal cell bodies (i.e., neuropil) was higher in layers 1–3 than in deeper layers (Figures 2A and 2B). In the HF of the same cases fixed in the same manner and cut at the same thickness, immunoreactivity in neuronal cell bodies, primary dendrites, and neuropil was distinctly higher than in the pSTG even when sections from these two areas were reacted together in the same experiment (cf. Figures 2A and 2B with 2C and 2D). This was most notable in CA3 and the dentate gryus (DG), where very high levels of dysbindin-1 occurred in large neurons (pyramidal cells in CA3 and hilar polymorph cells in the DG) and in synaptically dense layers of neuropil (strata oriens and radiatum in CA3 and the inner molecular layer in the DG: cf. Figures 1C and 2C and 2D (see also [5], [16], [43]). In both pSTG and HF, dysbindin-1 was also expressed at lower levels in at least a subset of smaller neurons (Figure 2B), probably interneurons, in both the pSTG and HF.

### Dysbindin-1 isoforms in synaptosomes

**Figure 3:**
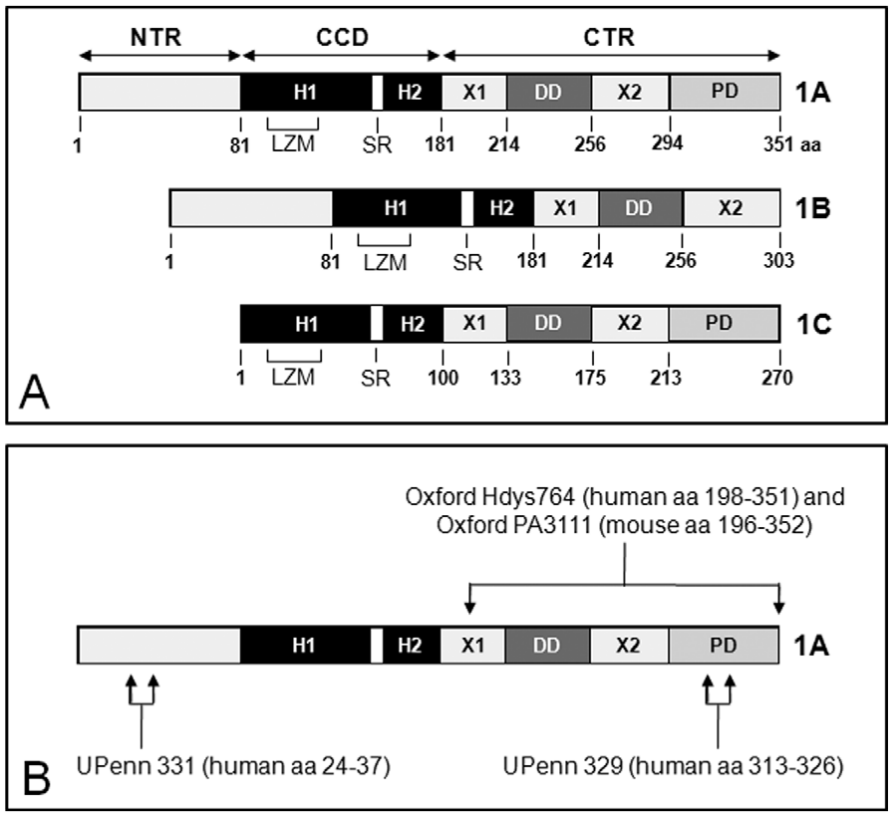
Major dysbindin-1 isoforms in humans and the immunogens used to generate antibodies to them by our research group. **A**: Schematic of the primary structure and protein segments of the three major dysbindin-1 isoforms in humans as characterized by Talbot et al. [5]. The numbers below isoforms designate amino acid (aa) location starting at the N-terminus. CCD = coiled coil domain composed of helices 1 and 2 (H1 and H2) separated by a stutter region (SR), CTR = carboxy terminus region, DD = the dysbindin domain, LZM = leucine zipper motif (LZM), NTR = amino terminus region, PD = PEST domain, X1 and X2 are uncharacterized regions. **B**: Location of immunogens for PA3111A, UPenn 331, and UPenn 329 shown on dysbindin-1A. Dysbindin-1A is 351 aa in humans, but 352 aa in mice.

As noted earlier, there are three major dysbindin-1 isoforms [5]. Figure 3A indicates the length and composition of these isoforms. All have a coiled coil domain (CCD, divided into helixes H1 and H2) important for certain protein-protein interactions. Isoform A (∼50 kDa: 351 amino acids [aa] in humans, 352 aa in mice) is full-length dysbindin-1 and has a PEST domain at its C-terminus. Isoform B (∼37 kDa: 303 aa in humans, not present in mice) differs from isoform A only in its C-terminus, which is shorter and lacks a PEST domain. Isoform C (∼33 kDa: 270 aa in humans, 271 aa in mice) differs from isoform A only in the absence of the N-terminus region found in the other isoforms in front of the CCD.

The specificity of our four dysbindin-1 antibodies (Oxford Hdys764, Oxford PA3111, UPenn 329, and UPenn 331: Figure 3B) was confirmed in tests using both positive and negative controls (see Text S1 and Figures S1 and S2). These tests also showed that PA3111and UPenn 331 have complementary abilities detecting dysbindin-1 isoforms (Table 1). PA3111 has a high affinity for both dysbindin-1A and -1C but a lesser affinity for dysbindin-1B, whereas UPenn 331 has a high affinity for dysbindin-1B but low affinity for dysbindin-1A and does not recognize dysbindin-1C (Figure S1). The present study is thus based on findings with those two antibodies. Hdys764 and UPenn 329 were not used for final testing since they yield reliable signals in postmortem tissue only at higher concentrations and hence with higher levels of background reactivity.

**Table 1:** Relative isoform affinity of dysbindin-1 antibodies tested.

Antibody	Dysbindin-1A	Dysbindin-1B	Dysbindin-1C
*Hdys764*	++++	—	++++
*PA3111*	++++	+/++	++++
*UPenn 329*	++++	∼	+++
*UPenn 331*	+	++++	—

Antibody affinities are based on Western blotting tests of whole tissue lysates from normal human brain tissue (caudate nucleus, dorsolateral prefrontal cortex, HF, and pSTG). Results of such tests on the caudate nucleus and pSTG are described in Text S1 and illustrated in Supplementary Figure 1.

**Figure 4:**
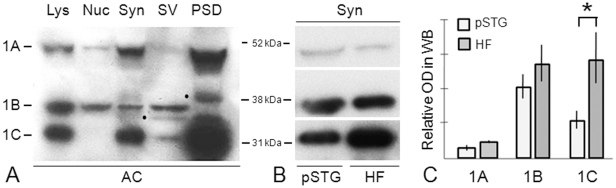
Concentration of dysbindin-1 isoforms A–C in synaptic tissue and relative synaptosomal levels of the isoforms in Western blots (WB) of normal human cerebral cortex and HF. **A**: Relative concentrations of the isoforms seen in whole cell lysates (Lys) and cell fractions: cell nuclei (Nuc), synaptosomes (Syn), synaptic vesicles (SV), and postsynaptic densities (PSD) using PA3111. While the samples were from the anterior cingulate cortex (AC), the same distribution pattern was seen in the pSTG and HF, except for lower synaptosomal dysbindin-1A. Bands marked to the left by dots mark likely dysbindin-1 degradation products in SV and PSD fractions. **B**: Relative concentrations of the dysbindin-1 isoforms in synaptosomes in the pSTG and HF using PA3111 for isoforms 1A and 1C and UPenn 331 for isoform 1B. The higher signal for dysbindin-1B compared to that seen in **A** probably reflects the much higher affinity of UPenn 331 than PA3111 for this isoform (Figure S1). **C**: Relative levels of synaptosomal dysbindin-1 isoforms in pSTG and HF compared using normalized PA3111 and UPenn 331 data for all normal cases studied (13 for pSTG, 15 for HF). Dysbindin-1C concentrations in the HF were significantly greater than in the pSTG (p = 0.0002, marked by an asterisk [*]). OD = β-actin normalized optical density of bands in Western blots.

Consistent with our earlier fractionation and immunohistochemical studies on dysbindin-1 in mouse and human brain tissue [43], all three of its major isoforms were concentrated in synaptic tissue though high concentrations of isoform B were also found in cell nuclei (Figure 4A). This was seen at the level of synaptosomes in all brain areas studied (anterior cingulate cortex, dorsolateral prefrontal cortex, HF, pSTG, and caudate nucleus), especially when using UPenn 331 to detect dysbindin-1B (Figure 4B). It was found, however, that the relative amounts of the isoforms differed between and within brain areas. Synaptosomal dysbindin-1A was clearly less concentrated in the pSTG and HF than in the three other brain areas studied (cf. Figures 4A and 4B) as confirmed when levels of actin-normalized data were compared across brain areas. In the pSTG and HF, dysbindin-1A was also much less abundant than dysbindin-1C: 3.1 times less abundant in the pSTG and 6.4 times less abundant in the HF as assessed with PA3111 (Figure 4C). Levels of dysbindin-1B relative to other dysbindin-1 isoforms could not be determined, because none of our antibodies had high affinity for all three isoforms. While the pSTG and HF in normal cases did not differ in levels of dysbindin-1A, the mean levels of dysbindin-1B and -1C were higher in the HF than in the pSTG. As shown in Figure 4C, the differences approached significance in the case of isoform B (t = −1.97, p = 0.059) and were highly significant in the case of isoform C (t = −4.31, p = 0.0002). Such differences may explain the higher levels of dysbindin-1 immunoreactivity in dendritic stalks and neuropil of the HF compared to the pSTG (see Figure 2).

### Distribution of dysbindin-1 isoforms in subsynaptic tissue fractions

**Figure 5:**
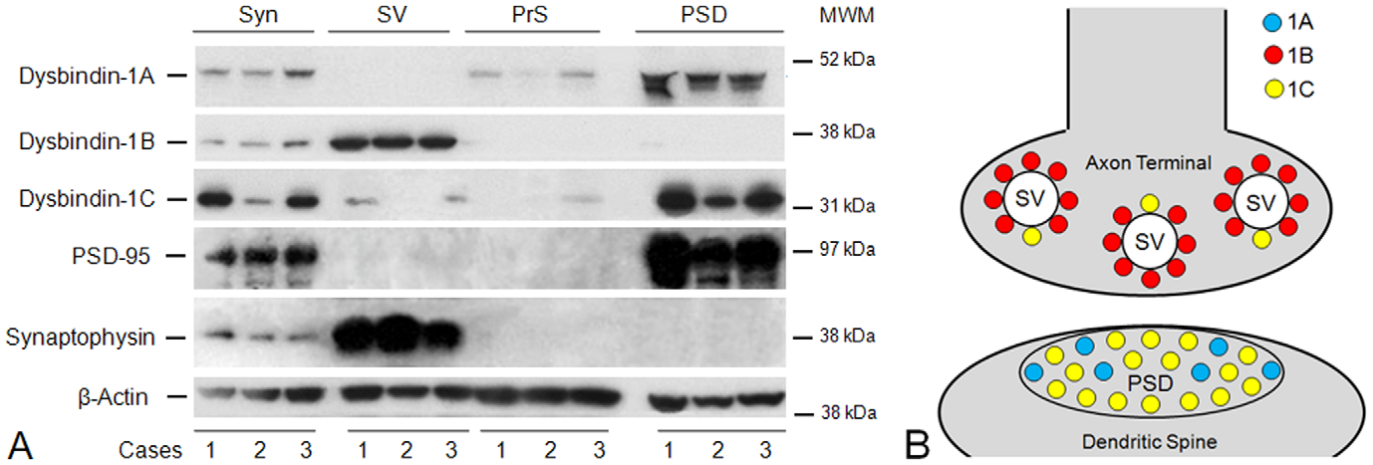
Subsynaptic localization of dysbindin-1 isoforms. **A**: Western blotting results on synaptosomal (Syn), synaptic vesicle (SV), presynaptic membrane (PrS), and postsynaptic density (PSD) fractions of the normal HF from three control cases (1–3) in this study. Successful synaptic fractionation was confirmed with synaptophysin as a marker for synaptic vesicles and PSD-95 as a marker for the PSD. No selective molecular marker is available for the PrS fraction, including syntaxin-1 since this proves to be ubiquitously distributed on subcellular membranes. β-actin served as the loading control. The blots were probed with PA3111 (1∶1000) for dysbindin-1A and -1C and with UPenn 331 (1∶5000) for dysbindin-1B. In synaptosomes, dysbindin-1A and -1C were found to be predominantly PSD proteins, while dysbindin-1B was found to be predominantly a synaptic vesicle-associated protein. MW = molecular weight marker position. **B**: Diagram of pre- and/or post-synaptic location of dysbindin-1 isoforms consistent with the Western blotting results just shown and with dysbindin-1 immunohistochemical findings at the electron microscopic level by Talbot et al. [43].

Each dysbindin-1 isoform was found to have a unique distribution pattern in synaptic tissue, one found in all tissues studied (anterior cingulate and dorsolateral prefrontal cortices, pSTG, HF, and caudate nucleus). This is shown for the isoforms in the normal human HF in Figure 5A, which presents Western blotting results on synaptosomes and subsynaptic fractions derived from them using a reliable fractionation method validated with electron microscopy and diverse pre- and post-synaptic markers by Phillips et al. [44], [45] in experimental animals and by our group in mouse and human brain tissue [43], [46], [47]. To show that the synaptic fractionation was successful, we include results with the markers of synaptic vesicles (synaptophysin) and postsynaptic densities (PSD-95).

Dysbindin-1A was undetectable in synaptic vesicle (SV) fractions, but was quite abundant in postsynaptic density (PSD) fractions. Conversely, dysbindin-1B was highly abundant in SV fractions, but virtually undetectable in PSD fractions. Dysbindin-1C was present to a small degree in the SV fractions of most cases, but was far more abundant in PSD fractions. Minor amounts of dysbindin-1A and -1C were detected in presynaptic membrane (PrS) fractions, but this may simply reflect incomplete separation of these soluble proteins from PSD fractions. A schematic summary of these findings is given in Figure 5B. The distribution pattern of dysbindin-1 isoforms in synapses was the same in normal and schizophrenia cases.

### Synaptosomal dysbindin-1 isoforms in schizophrenia cases vs. controls

**Figure 6:**
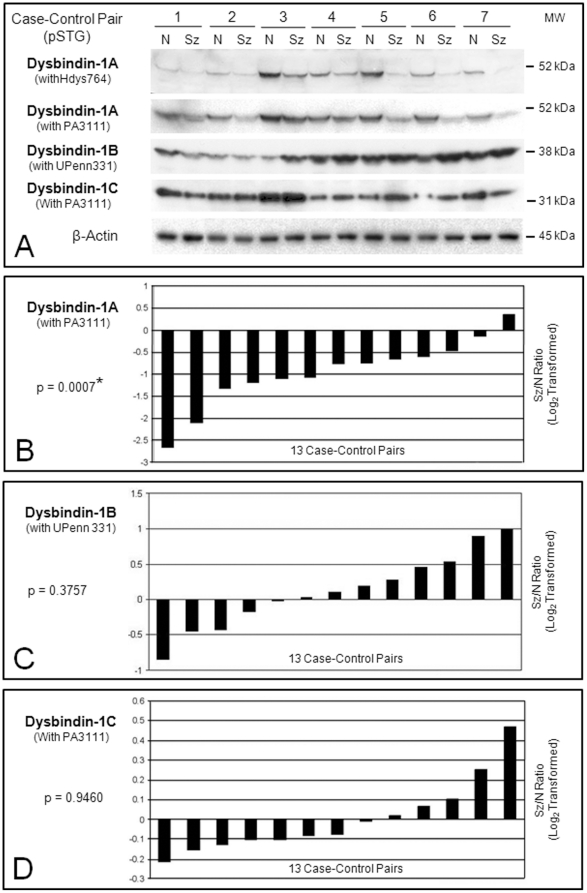
Relative levels of dysbindin-1A, -1B, and 1C in synaptosomal fractions of the pSTG from schizophrenia cases compared to their matched normal controls based on Western blotting results. **A**: Representative immunoblots on case-control pairs 1–7. Essentially the same results were obtained for dysbindin-1A with both antibodies against its CTR (i.e., Hdys764 [1∶800] and PA3111 [1∶1000]). Optical density readings confirmed that the bands in these Western blots were not close to saturation. **B–D**: Plotting of β-actin normalized data on dysbindin-1 isoforms for all 13 case-control pairs using PA3111 (1∶1000) or UPenn 331 (1∶5000). Each bar in these graphs gives the ratio of a dysbindin-1 isoform in a schizophrenia (Sz) case to that in its matched normal (N) control. The Sz/N ratios were log_2_ transformed so that negative values signify less dysbindin-1 isoform in the Sz case while positive values signify more of the isoform in the Sz case. Data for each Sz/N pair are not arranged according to the order of pairs shown in the Western blots, but simply in order of their ratios from maximum negative to maximum positive values. All 13 case-control pairs were used for statistical analyses on the three dysbindin-1 isoforms. Only dysbindin-1A showed significant differences between Sz cases and their matched controls (p = 0.0007). MW = molecular weight marker position.

**Figure 7:**
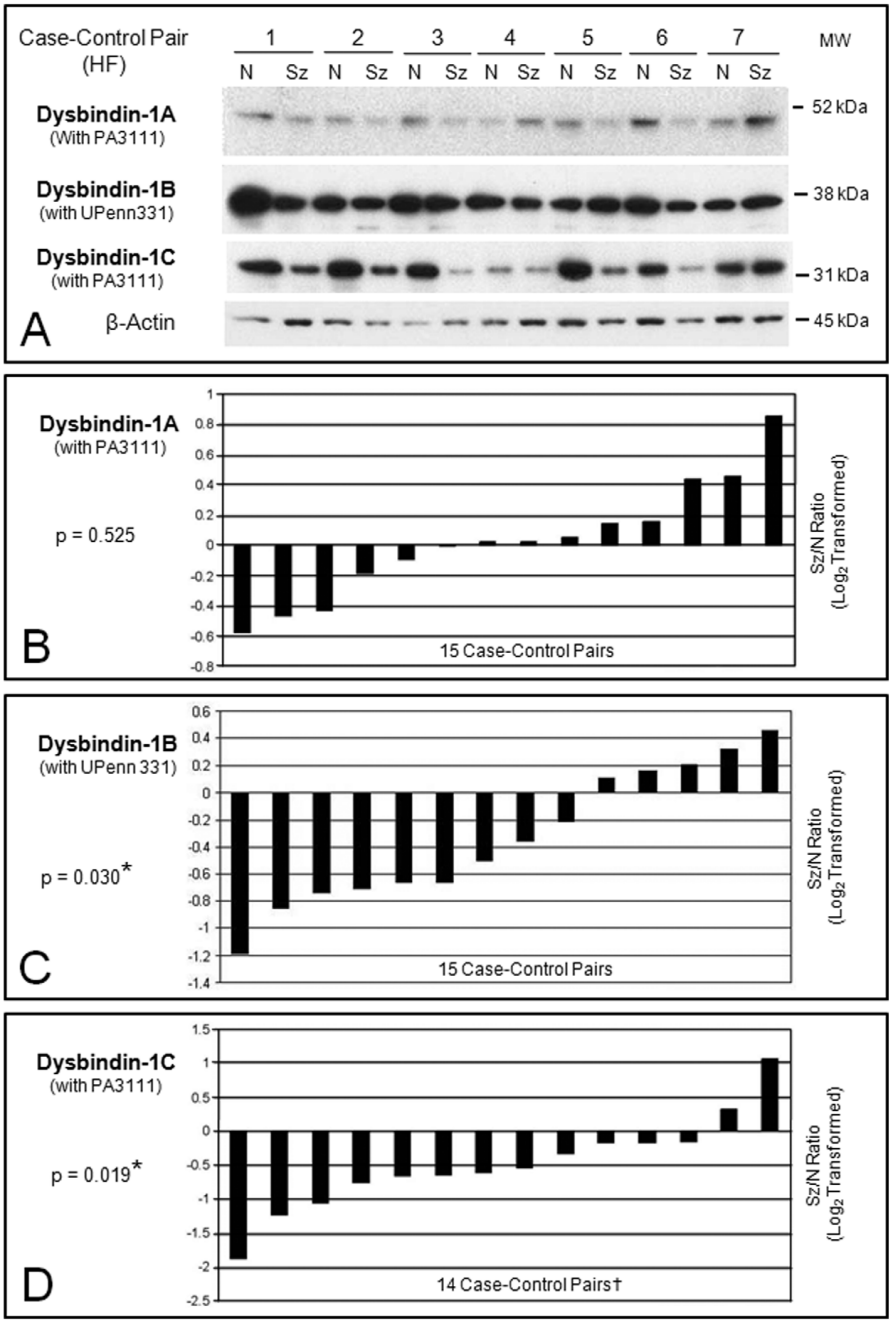
Relative levels of dysbindin-1A, -1B, and 1C in synaptosomal fractions of the HF from schizophrenia cases compared to their matched normal controls based on Western blotting results. **A**: Representative immunoblots on case-control pairs 1–7. Optical density readings confirmed that the bands in these Western blots were not close to saturation. **B–D**: Plotting of the β-actin normalized data on dysbindin-1A, -1B, and -1C for the 14–15 case-control pairs. Further explanation of the graphs is given in the caption to Figure 6. Significant differences were found for dysbindin-1B (p = 0.030) and -1C (p = 0.019). PA3111 was used at 1∶1000, UPenn 331 at 1∶5000. †Dysbindin-1C analyses omitted one case-control pair since the value for the control case was two SDs below the mean for the other controls. All 15 case-control pairs were used for the statistical analyses on dysbindin-1A and -1B. MW = molecular weight marker position.

Isoform-specific dysbindin-1 reductions occurred in synaptosomes of the schizophrenia cases, but the specific isoforms affected differed in the two brain areas studied. Figures 6A and 7A show representative Western blots of synaptosomal dysbindin-1 in the pSTG and HF of schizophrenia cases and their matched controls. Below the blots are graphs for each dysbindin-1 isoform. Each bar in these graphs indicates the ratio of a β-actin normalized isoform in a schizophrenia case compared to that in its matched control (i.e., the ratio for one case-control pair). The ratios are log_2_ transformed so that zero values indicate no differences between cases and controls, negative values indicate reduced dysbindin-1 in a schizophrenia case, and positive values indicate the reverse.

In pSTG (Figure 6), synaptosomal dysbindin-1A in pSTG was reduced in 12 of the 13 case-control pairs (92.30%) by an average of 47.73% (W = −87.00, p = 0.0007). Cases and controls did not differ significantly in synaptosomal levels of dysbindin-1B or 1C in this brain area.

In the HF (Figure 7), the results were opposite those in pSTG. No significant case-control differences were found in the HF for synaptosomal dysbindin-1A, but synaptosomal dysbindin-1B was reduced in 10 of the 15 case-control pairs (66.66%) by an average of 33.06% (W = −78.00, p = 0.0256), and synaptosomal dysbindin-1C was reduced in 12 of those 15 pairs (80.0%) by an average of 34.77% (W = −33.50, p = 0.0171). No significant correlations were found between the case-control ratios for dysbindin-1B and -1C.

### Consideration of potential confounding variables

The observed reductions in synaptosomal dysbindin-1 isoforms were not attributable to non-diagnostic variables (i.e., age, sex, PMI, pH), none of which were significantly correlated with levels of the dysbindin-1 isoforms tested. This is consistent with the design of this study. Cases and controls were matched for age (within 5 years), sex, and hemisphere studied. While availability of frozen tissue necessitated using largely different sets of cases for samples of the pSTG and HF, the normal and schizophrenia cases within and between these sets did not differ significantly in age, PMI, or brain tissue pH. In both sets of cases, the average PMIs were fairly short (9.5–11.0 h), and the average pHs were close to neutral (6.5–6.6) (Table 2). The fact that the same number of bands was seen in Western blots of the schizophrenia cases argues against lesser integrity of dysbindin-1 in those cases compared to controls, consistent with reports that many brain proteins remain intact under cool conditions within the PMIs of our cases (e.g., [48]).

**Table 2:** Demographic and autopsy data on cases studied.

Variable	pSTG		HF	
	Control	Schizophrenia	Control	Schizophrenia
*Number of Cases*	13	13	15	15
*Males/Females*	4/9	4/9	8/7	8/7
*Caucasians/African-Americans*	10/3	13/0	12/3	15/0
*Mean Age ± SD (y)*	79.17±7.60	80.0±5.79	80.20±9.37	81.33±6.94
*Hemisphere Sampled (L/R)*	7/6	7/6	8/7	8/7
*Mean PMI ± SD (h)*	9.51±4.62	10.92±3.16	9.60±7.10	11.03±4.08
*Mean Brain Tissue pH ± SD*	6.58±0.39	6.60±0.16	6.51±0.47	6.50±0.27

None of the subjects had histories of neurological or psychiatric conditions other than schizophrenia, and none displayed neuropathological abnormalities such as gross cell loss, infarcts, or unusually high densities of amyloid plaques, neurofibrillary tangles, or Lewy bodies. Nor were significant correlations found between antipsychotic dosages of our schizophrenia cases (expressed in chlorpromazine equivalents) a month prior to death and dysbindin-1 isoform levels, a finding in accordance with an earlier study by our group [16]. This is consistent with studies demonstrating that chronic haloperidol administration in mice has no significant effect on dysbindin-1 gene [49] or protein [16] expression.

Finally, our findings were not attributable to loss of synapses in the schizophrenia cases for three reasons. First, protein assay data on all synaptosomal samples were used to load the same amount of synaptic protein in the Western blots. Second, to correct for any loading errors, the dysbindin-1 isoforms levels analyzed were those normalized to synaptosomal levels of β-actin in each sample. Third, the synaptosomal fractions of our normal and schizophrenia cases showed no significant differences in either the synaptic vesicle marker synaptophysin (p>0.5) or the postsynaptic marker PSD-95 (p>0.9). The lack of differences between normal and schizophrenia cases in synaptophysin was observed previously in our quantitative immunohistochemical study of dysbindin-1 in the HF [16].

## Discussion

The present study demonstrates (1) that the three major dysbindin-1 isoforms are synaptically located in the human brain, (2) that they are differentially distributed in synaptic compartments, (3) that one or more of these pre- and/or post-synaptically localized isoforms are significantly reduced in synaptosomes of the pSTG and HF in schizophrenia without loss of synaptic vesicle or PSD markers. Each isoform was found to be associated primarily (if not exclusively) with either SVs or PSDs across brain areas in both normal and schizophrenia cases with dysbindin-1A limited to PSD fractions, dysbindin-1B to synaptic vesicle fractions, and dysbindin-1C primarily to PSD fractions. Dysbindin-1 isoform reductions observed in synaptosomes of schizophrenia cases thus indicate the subsynaptic compartments where those reductions occurred. We focus here on the implications of such reductions in the pSTG (BA 42 and caudal BA 22) and HF for the pathology and cognitive features of schizophrenia.

### Auditory cortices

In the pSTG, which consists largely of the auditory cortices [18]–[21], about 92% of our schizophrenia cases showed dysbindin-1A reductions averaging about 48% compared to their matched controls. This highly significant reduction (p = 0.0007) occurred without significant changes in levels of synaptic dysbindin-1B or -1C. Given our finding that synaptic dysbindin-1A is essentially limited to PSD fractions, these results indicate that an isoform-specific decrease in postsynaptic dysbindin-1 in auditory cortices occurs in schizophrenia. Our data do not establish the type(s) of neuron in which this decrease occurs, but the type likely to be affected the most are those expressing the highest levels of cellular and dendritic dysbindin-1, namely the pyramidal cells in layer III. They are characterized not only by their cell body shape, but also by their dendritic spines and axon collaterals terminating locally and in other auditory cortices (see [27]). In schizophrenia, Sweet and his colleagues [27] report that deep layer III of the primary and secondary auditory cortices in schizophrenia cases display dendritic spine density reductions of 27.2% and 22.2%, respectively. This was correlated with decreased densities of axon terminals in primary, but not in the secondary auditory cortex [26]. In light of the relevant literature, Sweet et al. [27] proposed that reduced excitatory afferent input on the dendritic spines of deep layer III pyramidal cells in auditory cortices may account for their decreased density in schizophrenia.

Decreased postsynaptic dysbindin-1A may be another factor promoting dendritic spine loss in auditory cortices of schizophrenia cases, potentially explaining why that occurs in secondary auditory cortex (BA 42) without axon terminal loss there. Reduced spine density is observed in mice with a cerebrocortical knockout of the gene encoding NMDA receptor subunit 1 [50], mRNA expression of which is reduced in sandy mice lacking dysbindin-1 due to a deletion mutation in *Dtnbp1* [51]. Spine density may also be affected by dysbindin-1 reductions in another way. Two proteins binding dysbindin-1 directly (rab11A [52]) or indirectly via pallidin (syntaxin-13, cf. [53], [54]) are known components of recycling endosomes whose membranes are required for growth and maintenance of dendritic spines [55]. These are highly labile structures [56] receiving most of the excitatory input on spiny neurons in the brain [57].

By lowering NMDA receptor densities or by disrupting endosomal trafficking in dendritic spines as recently postulated to occur in schizophrenia [58], postsynaptic dysbindin-1A reductions may impair excitatory transmission among auditory cortices and consequently promote dysfunctional connectivity among cerebrocortical areas in schizophrenia [59], which is thought to account for reduced gamma band synchrony in that disorder [60]. It is thus possible that postsynaptic dysbindin-1A reduction in auditory cortices in schizophrenia contributes to the many abnormalities in auditory information processing in the disorder [61], [62], including auditory hallucinations whose frequency and severity are significantly associated with volume reductions of the STG [39]–[42]. While we failed to detect PSD-95 reductions expected with spine loss in pSTG samples of schizophrenia cases, such a loss may have been masked by the inclusion of tissue beyond areas of the auditory cortices where spine loss has been shown in such cases (i.e., layer III of Brodmann areas 41 and 42 [27]).

### Hippocampal formation

In contrast to the pSTG, the HF in our schizophrenia cases showed normal levels of synaptic dysbindin-1A but reduced levels of synaptic dysbindin-1B and -1C. 67% of the cases showed decreased synaptosomal dysbindin-1B averaging 33% of controls (p = 0.026), while 80% showed decreased synaptosomal dysbindin-1C averaging 35% of controls (p = 0.017). It is unclear if the pair-wise (i.e., case-control) reductions in these two isoforms result from the decreased *DTNBP1* gene expression found in this brain area of schizophrenia cases by Weickert et al. [63]. Decreased dysbindin-1C levels have been found in the dorsolateral prefrontal cortex without altered *DTNBP1* gene expression [17]. An alternative possibility is suggested by the fact that the pair-wise reductions in dysbindin-1B and -1C in the HF were not correlated with each other. This may reflect differential posttranslational regulation of the two isoforms, perhaps due to the E3 ubiquitin ligase TRIM32, a dysbindin-1 binding partner known to ubiquitinate it [64]. In synaptosomes from the same HF samples used in the present study, we have found that levels of TRIM32 are inversely and significantly correlated with levels of dysbindin-1C, but not with levels of dysbindin-1B [65].

Given that synaptic dysbindin-1B is limited to SV tissue fractions, its reduction in the HF indicates a presynaptic decrease in HF dysbindin-1. This confirms our earlier immunohistochemical report [16] that the HF in schizophrenia suffers a reduction in presynaptic dysbindin-1. Indeed, the sample of schizophrenia cases in the present work is the third displaying this phenomenon, the previous two being another sample from the University of Pennsylvania and one from the Stanley Foundation.

Our previous immunohistochemical study [16] showed that the presynaptic dysbindin-1 reductions occur in axon terminals of intrinsic glutamatergic pathways of the HF, where our later fractionation and electron microscopic work found dysbindin-1 to be closely associated with synaptic vesicles [43]. Several groups have shown that reduced presynaptic dysbindin-1 impairs glutamatergic transmission [66]–[69].

HF reduction in synaptic dysbindin-1C could reflect decreases in both pre- and post-synaptic levels of the protein, but the latter would be predominant given that the bulk of this isoform is found in PSD fractions of synaptosomes. Such a postsynaptic effect was not detected in our earlier immunohistochemical report [16], which could not distinguish between dysbindin-1 in synaptic vs. non-synaptic portions of dendrites. At the electron microscopic level, we later localized dysbindin-1 in thick (i.e. glutamatergic) PSDs of dendritic spines in the HF of mice and macaques using electron microscopy [43]. Decreased dendritic spine density in much of the HF is suggested in schizophrenia by decreased expression of spinophilin mRNA [70] and confirmed in Golgi studies on the subiculum [71]. Reduced dysbindin-1C may contribute to spine loss in the same manner proposed above for reduced dysbindin-1A in auditory cortices.

By affecting pre- and post-synaptic aspects of glutamatergic transmission, the observed reductions in dysbindin-1 isoforms in the HF of schizophrenia cases could disrupt information processing in that structure and thus impair its cognitive functions. This is supported by studies on dysbindin-1 mutant (i.e., sandy) mice whose behavioral abnormalities resemble those of rodents with HF lesions [72]. The abnormalities include diverse cognitive deficits [reviewed by ref. 73], among which are spatial memory deficits [72], [74] common in schizophrenia [75], [76]. Since cognitive deficits form a core feature of that disorder [75], [77]–[79], the isoform-specific dysbindin-1 reductions we have found in schizophrenia may contribute to the clinical expression of schizophrenia.

## Materials and Methods

### Ethics statement

The present research on archived tissue samples taken at autopsy was approved by an Institutional Review Board at the University of Pennsylvania. Autopsy consent from next-of-kin or legal guardian was obtained in all cases. For most cases, consent was granted in writing before death and always confirmed after death. To keep postmortem delays to a minimum when written consent had not been obtained before death, verbal consent was obtained as witnessed by a third party and documented by the physician making the request. Written records of the consent for autopsy were archived. These procedures for written and verbal consent are standard medical practice in the U.S.A.

### Research design and subjects

A matched-pairs design was used in which each schizophrenia case was matched to a normal control in sex, age (within 5 y), and hemisphere sampled. The schizophrenia cases had participated in a longitudinal study of prospectively diagnosed subjects that conducted by the Schizophrenia Research Center at that university [80]. They met the diagnostic criteria for schizophrenia in DSM-IV (Diagnostic and Statistical Manual of Mental Disorders, 4th ed. [81]) as determined in consensus conferences after review of medical records, direct clinical assessments, and interviews of care providers. None of the cases had alternate or ambiguous DSM-IV diagnoses nor a history of substance abuse, neurological disorders (e.g., Alzheimer's disease, epilepsy, and Parkinson's disease), or acute neurological insults (anoxia, strokes, traumatic brain injury). Neither the controls nor schizophrenia cases were on ventilators near time of death.

### Tissue collection

After death, cases were stored at 2–4°C until transport to the University of Pennsylvania, where all autopsies were performed. Upon sagittal bisection, a hemispheres from each case was cut into coronal slabs, which were frozen overnight at −80°C and then sealed in plastic bags for long-term storage at −80°C. The pSTG and HF were later dissected from these coronal slabs, as were the anterior cingulate gyrus, dorsolateral prefrontal cortex, and caudate nucleus used for comparative purposes and for antibody testing.

Due to constraints imposed by frozen tissue availability, the subjects sampled for study of the pSTG (n = 26) were largely different than those sampled for study of the HF (n = 30). Tissue was specifically collected from BA 42 and caudal BA 22 in the pSTG and from intermediate rostrocaudal levels of the HF (dentate gyrus + hippocampal fields CA1-3 + subiculum) located as depicted in Figure 1. A summary of demographic and autopsy information on the two sets of cases studied is given in Table 2. The additional tissue taken from the anterior cingulate gyrus (n = 5), dorsolateral prefrontal cortex (n = 5) and the caudate nucleus (n = 4) for comparative purposes and antibody testing came from the control cases sampled for the pSTG or HF.

### Neuropathological assessment

The pSTG and HF were dissected at autopsy from the hemisphere not reserved for fresh freezing. This tissue was fixed in neutral-buffered formalin for 12–24 h, embedded in paraffin, and sectioned coronally at 6 µm. Sections were stained with hematoxylin and eosin to detect cell loss and infarcts or were reacted immunohistochemically (see below) for amyloid plaques, neurofibrillary tangles, or Lewy bodies with antibody βA4_2332_ (1∶8000) to β-amyloid 1–42 [82], AT8 (1∶1000) to phosphorylated tau (Thermo Scientific, Rockford IL, USA), Ub-1 (1∶400) to ubiquitin (Invitrogen 13–1600, Camarillo, CA, USA), or LB509 (1∶1000) to α-synuclein (Abcam ab27766, Cambridge, MA). After gross examination at autopsy and microscopic study of histological and immunohistochemical preparations, cases were excluded if they showed evidence of infarcts, tumors, gross cell loss, or densities of plaques, tangles, or Lewy bodies abnormal for the age of the individuals.

### Dysbindin-1 antibodies

Four affinity-purified, polyclonal rabbit antibodies against dysbindin-1 were used: Oxford Hdys764, Oxford PA3111, UPenn 329, and UPenn 331 (protein concentrations = 1.1, 0.1, 0.2, and 1.4 mg/ml, respectively). The Oxford antibodies were generated in Dr. Derek Blake's laboratory at the University of Oxford as described earlier [43]. Hdys 764 was raised against amino acids (aa) 198–351 of human dysbindin-1A (NCBI accession number NP_115498). PA3111 was raised against aa 196–352 of mouse dysbindin-1A (NP_080048). UPenn 329 was generated for Drs. Talbot and Louneva at the University of Pennsylvania by Sigma Genosys (The Woodlands, TX, USA) against a synthetic peptide consisting of GGESPVVQSDEEEV ( = aa 313–326 in the C-terminus of human dysbindin-1A). UPenn 331 was also generated by Sigma Genosys against a synthetic peptide consisting of SDKSREAKVKSKPR ( = aa 24–37 in the N-terminus of human dysbindin-1A). The aa sequences used as immunogens for UPenn 329 and UPenn 331 share no significant sequence homology with any known proteins other than dysbindin-1 isoforms. Results of specificity tests on these antibodies are detailed in the Text S1 and Figures S1 and S2.

### Immunohistochemistry

To determine the normal localization of dysbindin-1 in the human HF and pSTG, 6 µm sections from 9 control cases were reacted immunohistochemically with the antibody PA3111 (1∶300) using the avidin-biotin-peroxidase protocol of Talbot et al. [16], which may be summarized as follows. Formalin-fixed, paraffin-embedded pSTG and HF tissue was sectioned at 6 µm, mounted on glass slides, and air-dried. After de-waxing, tissue sections were quenched for endogenous peroxidase activity and then subjected to heat-induced epitope retrieval in 1 mM EDTA in 0.1 M Tris buffer (pH 8.0). Following cooling and rinsing between steps, the sections were incubated in primary antibody overnight at 4°C, then incubated in biotinylated secondary antibody for 1 h at room temperature, and subsequently treated with an avidin-biotin-peroxidase complex for 1 h before reaction with a diaminobenzidine (DAB) - hydrogen peroxide solution for 10 min. To visualize dysbindin-1 in human pSTG and HF, 6 µm coronal sections from 9 normal cases were reacted as just described with PA3111 (1∶300) validated in previous studies [5], [16], [17], [43] followed by light silver intensification of the DAB reaction product as detailed elsewhere [16]. No signal amplification was used in screening cases immunohistochemically for neuropathological conditions in the pSTG and HF with the antibodies for β-amyloid, tau, α-synuclein, and ubiquitin noted above (see Neuropathological Assessment).

### Preparation of tissue lysates, synaptosomes, and subsynaptic fractions

Whole-tissue lysates for antibody testing were prepared with 0.3 g of fresh frozen tissue from normal humans by homogenization in RIPA buffer (Sigma Aldrich, St. Louis, MO, USA, R0278) with a protease inhibitor cocktail (Sigma Aldrich P8340) and 2 µM EDTA. The lysate was then centrifuged at 15,000 *g* for 5 min at 4°C, and the supernatant collected for storage at −80°C. Nuclear extracts were prepared with the NXTRACT CelLytic NuCLEAR extraction kit (Sigma Aldrich) per the manufacturer's instructions.

Synaptosomes were obtained using a method based on Phillips et al. [44], [45]. For each case, 0.5 g fresh frozen tissue was homogenized in a sucrose solution (0.32 mol/L sucrose, 0.1 mmol/L CaCl_2_, 1 mmol/L MgCl_2_) containing a protease inhibitor cocktail (P8340, Sigma Aldrich). The homogenate was brought to a final sucrose concentration of 1.25 mol/L by adding 2 mol/L sucrose and 0.1 mmol/L CaCl_2_. After being overlaid with 1.0 mol/L sucrose and 0.1 mmol/L CaCl_2_, the homogenate was centrifuged at 100,000 *g* for 3 h at 4°C. The synaptosomal (Syn) fraction, which forms a band at the 1.25/1.0 mol/L sucrose interface, was collected and stored at −80°C for further fractionation or Western blotting. For the latter, 500 µl of the collected Syn material was washed twice in 0.1 mmol/L CaCl_2_ and stored in Laemmli loading buffer.

Subsynaptic fractions of synaptosomes were obtained using the method of Phillips et al. [44], [45] as adapted by Louneva et al. [46]. Synaptosomes from normal humans were accordingly washed twice in 0.1 mmol/L CaCl_2_ and then pelleted at 40,000 *g*. The pellets were solubilized in 20 mmol/L Tris-HCl, pH 6.0, 1% Triton X-100, and 0.1 mmol/L CaCl_2_, incubated on ice for 30 min, and centrifuged at 40,000 *g*. The supernatant is the synaptic vesicle (SV) fraction. The remaining pellet contains the synaptic junction consisting of pre- and post-synaptic membranes with the PSD. This pellet was solubilized in 20 mmol/L Tris-HCl, pH 8.0, 1% Triton X-100, and 0.1 mmol/L CaCl_2_, then incubated on ice for 30 min and centrifuged at 40,000 *g*. The resulting supernatant contains the PrS fraction, while the pellet contains the PSD fraction. SV and PrS fractions were acetone precipitated. All fractions were dissolved in 5% SDS. Protein concentration was determined by the BCA method (Pierce Chemical, Rockford, IL, USA).

### Western blotting

Synaptosomal samples were denatured in Laemmli loading buffer. After 5 min of boiling, the denatured samples were centrifuged at 10,000 g for 5 min. Each lane was loaded with 20 µg of total protein, because pilot tests on our samples showed that the ECL Plus chemiluminescence system (GE Healthcare, Waukesha, WI) used in this study detects dysbindin-1 isoforms reliably with that amount of protein. While two blots were needed to accommodate all normal and schizophrenia samples from one brain area, samples from any one matched pair of control and psychiatric cases were run in adjoining lanes on the same blot, and all blots for samples from any one brain area were imaged on the same film. All samples were run at least twice in separate replication experiments.

Proteins were separated by SDS-polyacrylamide gel electrophoresis on precast 12% Tris-glycine 1.5 mm gels (Invitrogen Novex, Carlsbad, CA, USA) and thereafter transferred onto a polyvinylidene difluoride membrane (Bio-Rad, Hercules, CA, USA) using a Novex XCell II Blot Module at 34 V for 80 min. Membranes were blocked for 1 h with 5% milk and incubated overnight at 4°C with primary antibody diluted in Tris-buffered saline with a final concentration of 3% milk and 0.1% Tween-20.

Primary antibodies were used at dilutions that allowed detection of their targets in a linear quantitative range: β-actin (Sigma Aldrich A3853 at 1∶4000), dysbindin-1A and 1C (Hdys764 at 1∶800; PA3111 at 1∶800–1∶1000, or UPenn 329 at 1∶200), dysbindin-1B (UPenn 331 at 1∶1000–1∶5000), PSD-95 (US Biological, Swampscott, MA, USA, P9150-10 at 1∶1000), and synaptophysin (Millipore, Billerica, MA, USA, MSB5258 at 1∶5000). After exposure to a primary antibody, membranes were incubated with a horseradish peroxidase-coupled secondary antibody (HRP-conjugated mouse IgG, GE Healthcare NA931) for 1 h at room temperature and reacted with the ECL Plus chemiluminescence system except β-actin blots reacted with a standard ECL chemiluminescence system (GE Healthcare) to avoid saturation. Immunoblotting for β-actin as a loading control was only performed on membranes previously probed for dysbindin-1 and then stripped with the ECL protocol before reprobing for β-actin. High-range rainbow molecular weight markers were used (GE Healthcare RPN756E).

Band densities were quantified with the GS-800 calibrated densitometer (Bio-Rad) using Quantity One 4.6.3 analysis software (Bio-Rad). Equivalent band areas across samples were selected using the volume rectangle tool. The global background subtraction method was used to obtain the net optical density of the bands. This raw data was then normalized to total loaded protein by calculating a ratio of the net optical density of each dysbindin-1 isoform to that of synaptosomal β-actin. All statistical analyses were performed on the normalized data. Equivalent statistical results were found on original and replication experiments run with the same samples, as well as in group-wise analyses of the data (i.e., all normal cases vs. all schizophrenia cases using t-tests)

### Data analysis

The matched pairs design minimized problems comparing data across Western blots encountered with between-groups designs when the number of samples exceeds the number of loading lanes on a single gel. Samples from both members of a case-control pair were run side-by-side on the same gel and thus under the same conditions. While all pairs could not be run on a single gel, our interest was in a measure we and others (e.g., [83], [84]) have found to be stable across Western blots, namely ratios of the protein level in a schizophrenia case to that in its matched control. Using β-actin normalized data, then, the ratio of a dysbindin-1 isoform in a schizophrenia case to that in its matched control was calculated. These ratios were log_2_ transformed so that a zero ratio indicates no difference between a case and its control, a negative ratio indicates a decrease in the case, and a positive ratio indicates an increase in the case. While the Wilcoxon signed ranks test is commonly used to analyze differences within matched pairs, log transformation enables its use to analyze ratio data since the log of a ratio between two values X/Y = logX−logY. This test was accordingly used to assess significance of case-control differences in each dysbindin-1 isoform tested.

Unpaired t-tests were used for between-group statistical comparisons of (a) normal vs. schizophrenia demographic and postmortem variables and (b) dysbindin-1 isoform levels in the pSTG and HF of normal cases. Pearson correlation coefficients were used to test the relationship of case-control differences in one dysbindin-1 isoform versus those in other such isoforms and to test the relationship of dysbindin-1 isoform levels in schizophrenia cases to neuroleptic medication expressed in chlorpromazine equivalents a month prior to death. The p values reported are two-tailed; values less than 0.05 were considered significant.

## Supporting Information

Figure S1 **Characterization of dysbindin-1 antibodies used in this study.** **A**: Western blots of the same whole tissue lysates probed with dysbindin-1 antibodies PA3111 (1∶800), Hdys764 (1∶800), UPenn 329 (1∶200), or UPenn 331 (1∶1000). Lysates were made from samples of the posterior superior temporal gyrus (pSTG) and caudate nucleus (Cd) of normal humans. **B**: Western blots of tagged recombinant dysbindin-1A, either full length (FL) or the C-terminus region (CTR) alone, probed with UPenn 329 (1∶200) or UPenn 331 (1∶5000) with or without antibody preadsorption by the peptide immunogen. The FL protein was histidine-tagged mouse dysbindin-1A. The CTR peptide (the peptide immunogen for Hdys764) consisted of aa 198–351 in human dysbindin-1A tagged with histidine-, serine-, and thioredoxin. The tagged FL and CTR sequences are predicted to have molecular weights of 40 and 35 kDa, respectively, but run 10 kDa higher probably due to the highly acidic CTR (PI∼3.7) as found for other highly acidic proteins [7] and possibly also due to ubiquitination [6]. **C**: Western blot showing that UPenn 331 (1∶5000) is highly selective for dysbindin-1B. It does recognize dysbindin-1A in whole brain lysates in humans and mice, but has much higher affinity for dysbindin-1B in humans and recognizes no such isoform in mice, which do not express a transcript for that isoform [3]. MWM = molecular weight marker positions.
(TIF)

Figure S2 **Specificity of PA3111 verified in tests on whole tissue lysates from brains of three wild-type (WT) and three homozygous sandy (Sdy/Sdy) mice.** WT mice express two major dysbindin-1 isoforms (1A and 1C). These isoforms run at about 50 and 33 kDa; neither is detected in sdy/sdy mice. Another band at about 26 kDa (†) is seen in WT mice, but is probably a degradation product of the 33 kDa band. Two bands marked by asterisks (*) are cross-reacting proteins since they are just as strong in the sdy/sdy mice lacking dysbindin-1. They are not seen in samples made from small amounts of tissue (i.e., from individual brain areas such as the cerebral cortex, HF, or cerebellum).
(TIF)

Text S1 **Specificity tests on the dysbindin-1 antibodies.** This describes and discusses tests on our dysbindin-1 antibodies with positive and negative controls.
(DOC)
