# Expression of Concern: Synaptic Dysbindin-1 Reductions in Schizophrenia Occur in an Isoform-Specific Manner Indicating Their Subsynaptic Location

**DOI:** 10.1371/journal.pone.0301152

**Published:** 2024-03-20

**Authors:** The PLOS ONE Editors

Following publication of this article, concerns were raised regarding Figures 1, 2, 5 and 6.

In discussing this matter, the corresponding author stated that the original, uncropped scans underlying Figure 5 and Figure 6 are no longer available, but they provided cropped blot images for the panels of concern.

The issues in Figures 1, 2, and 6 have been resolved or dismissed:

- The microscopy panels in Figure 1C, Figure 2C, and Figure 2D appear similar to Figure 4B, Figure 4E, and Figure 4G in [2], respectively. The corresponding author clarified that these images in [1] and [2] were derived from the same tissue sections but with different labelling, and that the images were used in the *PLOS ONE* article to provide qualitative information about the areal distribution of dysbindin-1 in the human brain.
- In Figure 6A there appears to be a vertical discontinuity between lanes 2 and 3 of the β-Actin panel. Given the published figure’s image quality and the unavailability of the original blot image we cannot fully clarify this issue. However, this affects only 1 of 7 case-control pairs for which western blot data are shown in the figure and so the *PLOS ONE* Editors concluded that the issue does not critically impact the figure’s results.

The Figure 5 concerns remain unresolved:

- In Figure 5A when levels are adjusted to visualise the background, within-panel similarities were noted in the PSD-95 panel (in lanes 4 and 5) and the Synaptophysin panel (lanes 10–12).

The corresponding author commented that the indicated regions of the PSD-95 panel appear similar but not identical, but they agreed that there appears to be a duplicated region in the Synaptophysin panel (lane 10–12). The cropped images provided by authors in support of these panels (see S1 File) include only single instances of each region of concern.

The *PLOS ONE* Editors concluded that the cropped blot images provided post-publication for Figure 5A support the figure’s overall results but did not resolve the concerns about within-blot similarities in the published figure. Therefore, the *PLOS ONE* Editors issue this Expression of Concern.

## Supporting information

S1 File Underlying blot images for panels of concern in Figure 5 and Figure 6. (ZIP)
