# Supplementary figures and images for: Synaptic Dysbindin-1 Reductions in Schizophrenia Occur in an Isoform-Specific Manner Indicating Their Subsynaptic Location
Source: PLoS One. 2011 Mar 1;6(3):e16886. doi: 10.1371/journal.pone.0016886 (PMC3046962; doi:10.1371/journal.pone.0016886)

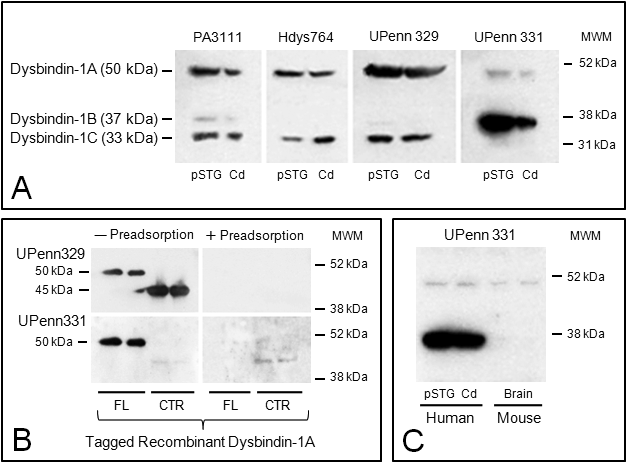

Supplement: Figure S1 — Characterization of dysbindin-1 antibodies used in this study. A: Western blots of the same whole tissue lysates probed with dysbindin-1 antibodies PA3111 (1∶800), Hdys764 (1∶800), UPenn 329 (1∶200), or UPenn 331 (1∶1000). Lysates were made from samples of the posterior superior temporal gyrus (pSTG) and caudate nucleus (Cd) of normal humans. B: Western blots of tagged recombinant dysbindin-1A, either full length (FL) or the C-terminus region (CTR) alone, probed with UPenn 329 (1∶200) or UPenn 331 (1∶5000) with or without antibody preadsorption by the peptide immunogen. The FL protein was histidine-tagged mouse dysbindin-1A. The CTR peptide (the peptide immunogen for Hdys764) consisted of aa 198–351 in human dysbindin-1A tagged with histidine-, serine-, and thioredoxin. The tagged FL and CTR sequences are predicted to have molecular weights of 40 and 35 kDa, respectively, but run 10 kDa higher probably due to the highly acidic CTR (PI∼3.7) as found for other highly acidic proteins [7] and possibly also due to ubiquitination [6]. C: Western blot showing that UPenn 331 (1∶5000) is highly selective for dysbindin-1B. It does recognize dysbindin-1A in whole brain lysates in humans and mice, but has much higher affinity for dysbindin-1B in humans and recognizes no such isoform in mice, which do not express a transcript for that isoform [3]. MWM = molecular weight marker positions. (TIF) [file pone.0016886.s001.tif]

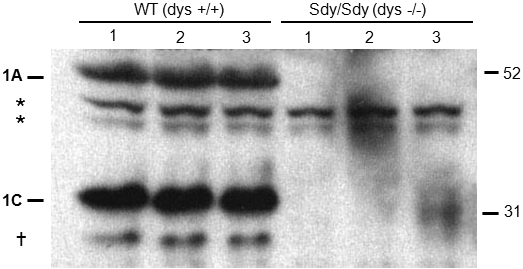

Supplement: Figure S2 — Specificity of PA3111 verified in tests on whole tissue lysates from brains of three wild-type (WT) and three homozygous sandy (Sdy/Sdy) mice. WT mice express two major dysbindin-1 isoforms (1A and 1C). These isoforms run at about 50 and 33 kDa; neither is detected in sdy/sdy mice. Another band at about 26 kDa (†) is seen in WT mice, but is probably a degradation product of the 33 kDa band. Two bands marked by asterisks (*) are cross-reacting proteins since they are just as strong in the sdy/sdy mice lacking dysbindin-1. They are not seen in samples made from small amounts of tissue (i.e., from individual brain areas such as the cerebral cortex, HF, or cerebellum). (TIF) [file pone.0016886.s002.tif]
