# Supplementary material for: Synaptic Dysbindin-1 Reductions in Schizophrenia Occur in an Isoform-Specific Manner Indicating Their Subsynaptic Location
Source: PLoS One. 2011 Mar 1;6(3):e16886. doi: 10.1371/journal.pone.0016886 (PMC3046962; doi:10.1371/journal.pone.0016886)
Supplement: Text S1 — Specificity tests on the dysbindin-1 antibodies. This describes and discusses tests on our dysbindin-1 antibodies with positive and negative controls. (DOC) [file pone.0016886.s003.doc]

Specificity Tests on Dysbindin-1 Antibodies

Using PA3111, a validated dysbindin-1 antibody [1-3] raised against the C-terminus region of isoform A (text Figure 3B), we detected bands at 50, 37, and 33 kDa in Western blots on whole-tissue lysates of all forebrain areas in all normal and schizophrenia cases tested. This included not only the posterior superior temporal gyrus (pSTG, n = 26) and hippocampal formation (HF, n = 30) on which this study was focused, but also anterior cingulate gyrus (n = 5), dorsolateral prefrontal cortex (n = 5), and caudate nucleus (n = 4) from normal cases This is illustrated in Figure S1A for a cerebrocortical area (pSTG) and a subcortical structure (the caudate nucleus). The three bands were not seen when the antibody had been preadsorbed with full-length dysbindin-1, as previously shown in tests of PA3111 on the HF [1,2]. The 50 kDa protein was readily identified as full-length dysbindin-1, previously reported to run at 48-53 kDa [1-5]. This exceeds the predicted 40 kDa mass of dysbindin-1A, probably attributable to its highly acidic C-terminus [3,4] and perhaps also to post-translational modifications such as phosphorylation [3] and ubiquitination [6]. The 37 and 33 kDa bands are very close to the predicted molecular weight of human dysbindin-1B and -1C (35 and 30 kDa, respectively).

As shown in Figure S1, the identity of the 50, 37, and 33 kDa bands seen with PA3111A were confirmed with three other polyclonal dysbindin-1 antibodies raised against peptide sequences specified in text Figure 1B. Two antibodies (Hdys764 and UPenn 329) were raised against sequences in the C-terminal regions of dysbindin-1A and 1C, regions which are identical in those isoforms but differ significantly from the C-terminus region of dysbindin-1B (see text Figure 3A). The third antibody (UPenn 331) was raised against the N-terminus region of dysbindin-1A and -1B, a region which is identical in those isoforms but is absent in dysbindin-1C (see text Figure 3A). Hdys764 and UPenn 329 accordingly recognized the 50 and 33 kDa bands, but not the 37 kDa band, while UPenn 331 recognized the 50 and 37 kDa bands, but not the 33 kDa band (Figure S1A).

Tests with recombinant proteins confirmed that whereas both UPenn 329 and UPenn331 recognize full-length dysbindin-1A, UPenn 331 does not recognize the C-terminus of that protein (Figure S1B). This supports the view that UPenn 331 selectively recognizes the N-terminus sequence (which served as its immunogen), preventing it from recognizing dysbindin-1 C, which lacks that N-terminus sequence. UPenn 331 also differed from the other antibodies in its low affinity for dysbindin-1A in the 50 kDa band and its high affinity for dysbindin-1B in the 37 kDa band (Figure S1A). This was verified in tests on mice, which have an ortholog of human dysbindin-1A and -1C, but not of dysbindin-1B [3,8]. In whole brain lysates of the mouse, UPenn 331 showed low affinity for a 50 kDa band, but recognized no other band (Figure S1C). These findings collectively indicate that UPenn 331 is a sensitive and largely selective antibody for dysbindin-1B. It thus provides data complementary to PA3111, which has a high affinity for dysbindin-1A and -1C but only low affinity for dysbindin-1B judging from the weak 37 kDa band seen Western blots (Figure S1A). Consequently, we relied upon PA3111A to detect dysbindin-1A and -1C, but UPenn 331 to detect dysbindin-1B.

Further evidence for the specificity of PA3111 was obtained in homozygous sandy mice, which lack dysbindin-1 as a consequence of a mutation in Dtnbp1 [3,8,9]. Tests on these mice and wild-type littermates showed that PA3111 recognized no proteins at the molecular weight of either dysbindin-1 isoform expressed in mice (i.e., isoforms A and C; Figure S2).

References

1. Talbot K, Eidem WL, Tinsley CL, Benson MA, Thompson EW, et al. (2004) Dysbindin-1 is reduced in intrinsic, glutamatergic terminals of the hippocampal formation in schizophrenia. J Clin Invest 113: 1353-1363.

2. Talbot K, Cho, D-S, Ong W-Y, Benson MA, Han L-Y, et al. (2006) Dysbindin-1 is a synaptic and microtubular protein that binds brain snapin. Hum Mol Genet 15: 3041-3054.

3. Talbot K, Ong WY, Blake DJ, Tang J, Louneva N, et al. (2009) Dysbindin-1 and its protein family with special attention to the potential role of dysbindin-1 in neuronal functions and the pathophysiology of schizophrenia. In: Javitt D, Kantrowitz J, editors. Handbook of neurochemistry and molecular neurobiology, 3^rd^ ed., vol. 27 (Schizophrenia). New York: Springer US. pp. 107-241.

4. Benson MA, Newey SE, Martin-Rendon E, Hawkes R, Blake DJ (2001) Dysbindin, a novel coiled-coil-containing protein that interacts with the dystrobrevins in muscle and brain. J Biol Chem 276: 24232-24241.

5. Tang J, LeGros RP, Louneva N, Yeh L, Cohen JW, et al. (2009) Dysbindin-1 in dorsolateral prefrontal cortex of schizophrenia cases is reduced in an isoform-specific manner unrelated to dysbindin-1 mRNA expression. *Hum Mol Genet* **18**: 3851-3863.

6. Locke M, Tinsley CL, Benson MA, Blake DJ (2009) TRIM32 is an E3 ubiquitin ligase for dysbindin. Hum Mol Genet 18: 2344-2358.

7. Zirwes RF, Schmidt-Zachmann MS, Franke WW (1997) Identification of a small, very acidic constitutive nucleolar protein (NO29) as a member of the nucleoplasmin family. Proc Natl Acad Sci USA 94: 11387-11392.

8. Talbot K (2009) The sandy (sdy) mouse: a dysbindin-1 mutant relevant to schizophrenia research. Prog Brain Res 179 (*Genetic Models of Schizophrenia*): 87-94.

9. Li W, Zhang Q, Oiso N, Novak EK, Gautam R, et al. (2003) Hermansky-Pudlak syndrome type 7 (HPS-7) results from mutant dysbindin, a member of the biogenesis of lysosome-related organelles complex 1 (BLOC-1). Nat Genet 35: 84-89.
