# Supplementary figures and images for: Expression of Concern: Synaptic Dysbindin-1 Reductions in Schizophrenia Occur in an Isoform-Specific Manner Indicating Their Subsynaptic Location
Source: PLoS One. 2024 Mar 20;19(3):e0301152. doi: 10.1371/journal.pone.0301152 (PMC10954157; doi:10.1371/journal.pone.0301152)

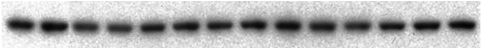

Supplement: S1 File — (ZIP) [file pone.0301152.s001.zip › S1 File/Talbot - PLoS One Actin in STG of Normal and Sz Cases (1).tif]

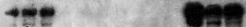

Supplement: S1 File — (ZIP) [file pone.0301152.s001.zip › S1 File/Talbot - PLoS One PSD 95 in Subsynaptic Fractions (4).tif]

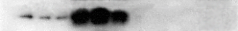

Supplement: S1 File — (ZIP) [file pone.0301152.s001.zip › S1 File/Talbot - PLoS One Synaptophisin in Subsynaptic Fractions (2).tif]
